# Contemporary medical therapy for heart failure across the ejection fraction spectrum: The OPTIPHARM‐HF registry

**DOI:** 10.1002/ejhf.70074

**Published:** 2025-12-17

**Authors:** Riccardo M. Inciardi, Maurizio Volterrani, Gianluigi Savarese, Muthiah Vaduganathan, Chiara Oriecuia, Carlo M. Lombardi, Cristina Gussago, Piergiuseppe Agostoni, Pietro Ameri, Giuseppe Armentaro, Chiara Arzilli, Nadia Aspromonte, Andrea Attanasio, Roberto Badagliacca, Lucia Barbieri, Pier Paolo Bocchino, Francesca Bursi, Matteo Cameli, Martino Canonero, Jeness S. Campodonico, Teresa Capovilla, Erberto Carluccio, Stefano Carugo, Vincenzo Castiglione, Dario Catapano, Manlio Cipriani, Michele Correale, Domenico D'Amario, Raffaele De Caterina, Gaetano M. De Ferrari, Emilia D'Elia, Luca Di Odoardo, Michele Emdin, Luigi Falco, Giulia Ferrante, Alessandra Fornaro, Paolo Fornaro, Gionata Guastamiglio, Marco Guazzi, Massimo Iacoviello, Massimo Imazio, Enrico Incaminato, Maria Teresa La Rovere, Sergio Leonardi, Marta Maccallini, Giulia E. Mandoli, Daniele Masarone, Marco Masetti, Alberto Mazzoni, Marta Mazzotta, Marco Merlo, Luigi Moschini, Filippo Novarese, Alberto Palazzuoli, Maria C. Pastore, Giuseppe Patti, Roberto F.E. Pedretti, Stefano Pidello, Massimo F. Piepoli, Giuseppe Pinto, Luciano Potena, Claudia Raineri, Filippo M. Rubbo, Mario Sabatino, Andrea Salzano, Angela Sciacqua, Michele Senni, Paolo Severino, Gianfranco Sinagra, Barbara Sposato, Stefano Taddei, Alessandro Valleggi, Carlo Vignati, Dario Vizza, Claudia Specchia, Giuseppe Rosano, Marco Metra, the OPTIPHARM‐HF investigators, Marianna Adamo, Marilisa Ambrosio, Paolo Biagioli, Valeria Borelli, Natale Brunetti, Margherita Calcagnino, Giuseppe Caminiti, Francesco Canonico, Angelo Caporotondi, Velia Cassano, Ali Chraim, Valentina Codazzi, Giuliana Cimino, Francesco Clemenza, Giuditta Cuccuru, Giulia De Lio, Giandomenico Disabato, Marcello Divino, Salvatore D'Isa, Roberta Famiani, Enrica Fede, Letizia Fiòorentino, Alberto Foa', Paolo Fornaro, Noemi Furlotti, Nicolò Gallingani, Alberto Genova, Stefano Ghio, Federico Gobbi, Daniela Grassano, Lucia Guerisoli, Manuela Iseppi, Simona Leone, Ferdinando Loiacono, Valeria Luberto, Rossella Manai, Niccolò Manetti, Lina Manzi, Fabio Marsico, Francesco Maruca, Gabriele Masini, Irene Mattavelli, Ciro Mauro, Alberto Mazzoni, Marta Mazzotta, Pierre Meynet, Valentina Morsella, Giulia Nemola, Martina Niccolai, Savina Nodari, Matteo Pagnesi, Gianpaolo Palmieri, Alberto Panza, Francesca Parisi, Carlo Alberto Pastura, Anna Lisa Picerni, Riccardo Pilia, Ilaria Pirrone, Nicola R. Pugliese, Claudio Puteo, Federica Ramani, Gloria Santangelo, Laura Scelsi, Edoardo Sciatti, Lorenza Sforzini, Giulia Spoto, Davide Stolfo, Roberto Tarantini, Maria Cristina Tavera, Floriana Tortomasi, Eduardo Valente, Giuseppe Varricchione, Enrico Vizzardi, Greta Zagni, Mattia Zampieri

**Affiliations:** ^1^ Institute of Cardiology, ASST Spedali Civili, Department of Medical and Surgical Specialties, Radiological Sciences, and Public Health, University of Brescia Brescia Italy; ^2^ Division of Cardiology, Department of Clinical Science and Education, Center for Heart Failure and Arrhythmia Karolinska University Hospital Stockholm Sweden; ^3^ Cardiovascular Division Brigham and Women's Hospital, Harvard Medical School Boston MA USA; ^4^ Department of Exercise Science and Medicine, San Raffaele Open University of Rome, Italy, and Cardiopulmonary Department, RCCS San Raffaele Roma Rome Italy; ^5^ Department of Molecular and Translational Medicine University of Brescia Brescia Italy; ^6^ Centro Cardiologico Monzino, IRCCS Department of Clinical Sciences and Community Health Milan Italy; ^7^ Department of Clinical and Community Medicine, University of Milan Milano Italy; ^8^ Cardiovascular Disease Unit, IRCCS Ospedale Policlinico San Martino Genoa Italy; ^9^ Department of Internal Medicine University of Genoa Genoa Italy; ^10^ Geriatrics Division. AOU R. Dulbecco, Department of Medical and Surgical Sciences University Magna Graecia of Catanzaro Catanzaro Italy; ^11^ Cardiology Division, Fondazione Toscana Gabriele Monasterio Pisa, Italy; Interdisciplinary Center for Health Sciences Scuola Superiore Sant'Anna Pisa Italy; ^12^ Department of Cardiovascular ad Thoracic Sciences, Fondazione Policlinico Universitario A. Gemelli IRCCS, Department of Cardiovascular and Pulmonary Sciences Catholic University of the Sacred Heart Rome Italy; ^13^ Clinical Cardiology, IRCCS Policlinico San Donato, San Donato Milanese Milan Italy; ^14^ Department of University of Wroclaw Wroclaw Poland; ^15^ Department of Clinical Internal, Anesthesiological and Cardiovascular Sciences Sapienza University of Rome, Policlinico Umberto I Rome Italy; ^16^ Department of Cardio‐Thoracic‐Vascular Diseases, Foundation IRCCS Ca' Granda Ospedale Maggiore Policlinico, Department of Clinical Sciences and Community Health University of Milan Milan Italy; ^17^ Division of Cardiology, Cardiovascular and Thoracic Department, Città della Salute e della Scienza Hospital, Department of Medical Sciences University of Turin Turin Italy; ^18^ Cardiology Department University of Milano, San Paolo Hospital Milan Italy; ^19^ Division of Cardiology, Department of Medical Biotechnologies University of Siena Siena Italy; ^20^ Cardiothoracovascular Department Azienda Sanitaria Universitaria Giuliano Isontina, University of Trieste Trieste Italy; ^21^ Cardiology and Cardiovascular Pathophysiology, Santa Maria della Misericordia Hospital, University of Perugia Perugia Italy; ^22^ Heart Failure Unit, Department of Cardiology AORN dei Colli‐Monaldi Hospital Naples Italy; ^23^ Mediterranean Institute for Transplantation and Advanced Specialized Therapies ISMETT‐IRCCS Palermo Italy; ^24^ Cardiology Unit, University Hospital Policlinico Riuniti, Department of Medical and Surgical Sciences University of Foggia Foggia Italy; ^25^ Division of Cardiology, AOU Maggiore della Carità, Department of Translational Medicine Università del Piemonte Orientale Novara Italy; ^26^ Cardiology Division, Cardiothoracic and Vascular Department, Pisa University Hospital, Department of Surgical, Medical and Molecular Pathology and of Critical Sciences University of Pisa Pisa Italy; ^27^ Cardiology Unit, Cardiovascular Department ASST Papa Giovanni XXIII Bergamo Italy; ^28^ Cardiology Unit Careggi University Hospital Florence Italy; ^29^ Department of Medicine (DMED), University of Udine, and Cardiothoracic Department University Hospital Santa Maria della Misericordia, ASUFC Udine Italy; ^30^ Department of Cardiology, Istituti Clinici Scientifici Maugeri IRCCS Montescano (PV) Italy; ^31^ Division of Cardiology, Fondazione IRCCS Policlinico San Matteo, Department of Molecular Medicine University of Pavia Pavia Italy; ^32^ Humanitas Clinical and Research Hospital IRCCS, Rozzano, Department of Biomedical Sciences Humanitas University Milan Italy; ^33^ Heart Failure and Transplant Unit, IRCCS Azienda Ospedaliero Universitaria di Bologna Bologna Italy; ^34^ Division of Cardiology Ospedale Oglio Po, ASST Cremona Casalmaggiore Italy; ^35^ Cardiovascular Diseases Unit, Cardiothoracic and Vascular Department Le Scotte Hospital, University of Siena Siena Italy; ^36^ Unit of Cardiology, Cardiovascular Department IRCCS MultiMedica, Sesto San Giovanni Milan Italy; ^37^ Cardiology Unit, AORN A. Cardarelli Naples Italy; ^38^ Department of Translational Medical Sciences, ‘Federico II’ University, Interdepartmental Center for Gender Medicine Research ‘GENESIS’, ‘Federico II’ University Naples Italy; ^39^ Department of Clinical and Experimental Medicine University of Pisa Pisa Italy; ^40^ Department of Medical Sciences, Centre for Clinical and Basic Research IRCCS San Raffaele Pisana Rome Italy

**Keywords:** Heart failure, Medical therapy, Optimization, Registry, Ejection fraction spectrum

## Abstract

**Aims:**

Despite guideline recommendations, guideline‐directed medical therapy (GDMT) remains underused and underdosed in patients with heart failure (HF) across the ejection fraction (EF) spectrum. The aim of this study was to evaluate GDMT use, dosing, and implementation in a contemporary, nationwide HF cohort.

**Methods and results:**

The OPTIPHARM‐HF (NCT06192524) is a prospective, multicentre, observational study enrolling adult patients with HF, across 32 Italian HF centres. Clinical characteristics, medical therapy prevalence and change after first visit have been assessed in patients with reduced (HFrEF: EF ≤40%), mildly reduced (HFmrEF: EF 40–49%), and preserved EF (HFpEF: EF ≥50%). From September 2022 to December 2024, 3054 patients (mean age 69 ± 12 years, 25% female) were enrolled: 56% with HFrEF, 21% with HFmrEF, and 23% with HFpEF. Among HFrEF, prescription frequencies were: 90% for beta‐blockers; 19% for angiotensin‐converting enzyme inhibitors (ACEi)/angiotensin II receptor blockers (ARB); 61% for angiotensin receptor–neprilysin inhibitors (ARNI); 72% for mineralocorticoid receptor antagonists (MRA); and 69% for sodium–glucose co‐transporter 2 inhibitors (SGLT2i). Less than 60% achieved ≥50% of target doses. Quadruple therapy was received by 47% of the patients. After first visit, there was an increase in prescription of all classes of drugs, and titration to quadruple therapy was attained in 64% (*p* < 0.001). Among HFmrEF, 88% were on beta‐blockers, 34% on ACEi/ARB, 49% on ARNI, 63% on MRA, and 59% on SGLT2i. In the HFpEF group, 76% were on beta‐blockers, 49% on ACEi/ARB, 18% on ARNI, 49% on MRA and 40% on SGLT2i. After the first visit, SGLT2i prescription significantly increased both in HFmrEF (74%, *p* < 0.001) and HFpEF (54%, *p* < 0.001).

**Conclusions:**

Use of GDMT remains suboptimal across the EF spectrum although the adoption of quadruple GDMT in HFrEF and of SGLT2i in HFmrEF and HFpEF increased in recent years.

## Introduction

Despite recent advances in its management, heart failure (HF) represents a major public health issue affecting more than 55 million people worldwide and is a leading cause of morbidity and mortality.^1^ Multiple pharmacological options are now available for HF patients across the left ventricular ejection fraction (LVEF) spectrum.^2^, ^3^, ^4^, ^5^ Contemporary combination and up‐titration of beta‐blockers, mineralocorticoid receptor antagonists (MRA), angiotensin receptor–neprilysin inhibitor (ARNI) sacubitril/valsartan, and sodium–glucose co‐transporter 2 inhibitors (SGLT2i), is recommended by international guidelines for HF with reduced ejection fraction (HFrEF) patients and may be considered for those with mildly reduced ejection fraction (HFmrEF), to improve survival and cardiovascular outcomes.^5^, ^6^ Recent advances have also been observed for patients with HF and preserved ejection fraction (HFpEF) since SGLT2i have shown to improve HF outcomes and quality of life and are recommended as a first‐line treatment.^5^, ^6^, ^7^ Despite proven benefits and strong recommendations to improve guideline‐directed medical therapy (GDMT), medication use and dosing in routine clinical practice is still lacking, and patients often remain undertreated for multiple reasons.^8^ As previously observed in registries and real‐world healthcare databases, cost/access limitations, absolute or relative contraindications, real or perceived intolerance and, likely, most of all, treatment inertia are key factors hampering GDMT implementation. These data largely characterized HFrEF populations prior to the introduction of SGLT2i and their evidence of benefit across the whole LVEF spectrum. Better understanding of updated current practice management, gaps in medication administration and barriers to GDMT implementation in a contemporary HF population across the LVEF spectrum, is critical to deliver better patient care. The OPTIimal PHARMacological therapy for patients with Heart Failure (OPTIPHARM‐HF; NCT06192524) registry offers the opportunity to study a contemporary national ‘real‐world’ HF population and to assess the extent to which current recommendations are adopted in clinical practice.^9^

## Methods

### Study design

The rationale and design of the OPTIPHARM‐HF registry has previously been described.^9^ Briefly, the OPTIPHARM‐HF is an ongoing prospective, observational, nationwide registry of adult patients with HF. The study population included patients with HF, as defined by current international guidelines, regardless of LVEF, aged 18 years or older, able to give their written informed consent to participate in the registry. Both outpatients and inpatients hospitalized with chronic HF or after an episode of decompensated HF were consecutively recruited. Key exclusion criteria included planned participation or participation in a clinical trial; life expectancy <1 year because of non‐cardiac causes; previous heart transplant or left ventricular assist device implantation; cardiac dysfunction in the absence of symptoms, i.e. pre‐HF, according to the universal definition of HF.^10^ The research protocol was approved by the ethics committee of the University Hospital of Brescia under the number NP5441 and at all sites and complied with the Declaration of Helsinki. All patients provided written informed consent.

### Data collection

Patients were enrolled (online supplementary *Figure* *S1*) across 32 Italian HF centres. A full list of participating sites is provided in online supplementary *Figure* *S2*. This primary report of the prospective registry describes the use of GDMT, in terms of both drug prescription and drug dosing, in patients with HFrEF, HFmrEF, and HFpEF.

Data collection is currently completed for the first visit (V1; visits are considered as outpatient visits or at‐discharge evaluation in hospitalized patients), while it is under collection for the ongoing prespecified visits per study protocol and longer‐term follow‐up. At V1 demographic, vital signs, main echocardiographic cardiac parameters and biochemistry data and medical history were collected. Detailed medication information included dosage, main causes of underuse and underdosing and therapeutic changes at the time of first visit and at the end. Drug dosing was classified into <50%, 50% to <100% and ≥100% target doses, according to European Society of Cardiology (ESC) guideline recommendations and previous studies. We applied similar cut‐off across the LVEF spectrum for HFmrEF and HFpEF patients.

### Statistical analysis

Continuous variables were shown as means and standard deviations, skewed variables as medians and interquartile ranges (IQR), dichotomous variables as counts and percentages. Between‐group comparisons for baseline characteristics were conducted using the two‐sample *t*‐test or the Wilcoxon rank‐sum test when appropriate. Comparisons involving three or more groups were conducted using ANOVA or the Kruskal–Wallis test when appropriate. The Chi‐squared test was used for between‐group comparisons of categorical variables, with Fisher's exact test applied when at least one expected count was less than 5. McNemar's test was used for within‐group comparisons of changes in medical therapy from baseline to the end of V1. The dose of each drug class was expressed as the mean percentage relative to the target dose. Data were calculated based on non‐missing values. The overall proportion of missing data was low, with most variables having minimal or no missing values except for N‐terminal pro‐B‐type natriuretic peptide (NT‐proBNP) (around 30%). A two‐tailed p‐value <0.05 was considered statistically significant. Statistical analyses were performed with SAS software (version 9.4; SAS Institute, Cary, NC, USA) and R version 4.3.1 (R Core Team 2024, R Foundation for Statistical Computing, Vienna, Austria).

## Results

### Baseline characteristics

Between September 2022 and December 2024, 3054 patients were enrolled. Mean age was 69.2 ± 12.3 years, 24.8% were female and 97.8% Caucasian. Mean LVEF was 40 ± 11.7%. Overall, 1720 (56.3%) patients were categorized as HFrEF, 625 (20.5%) as HFmrEF, and 709 (23.2%) as HFpEF. Patients in the higher ejection fraction categories were older, more frequently female, and exhibited a greater burden of comorbidities (e.g. hypertension, atrial fibrillation) alongside lower NT‐proBNP levels (*Table* *1*). Impaired kidney function was less common in HFmrEF compared with HFrEF and HFpEF, with the latter showing the highest prevalence of patients with estimated glomerular filtration rate <60 ml/min/1.73 m^2^. Patients with HFmrEF had similar demographic characteristics and comorbidities as those with HFrEF, but with lower prevalence of coronary artery disease and history of myocardial infarction and lower values of NT‐proBNP. Those with HFrEF were more frequently hospitalized for heart failure in the last 12 months before study enrolment as compared to HFmrEF and HFpEF.

**Table 1:** Patients' baseline characteristics according to LVEF categories

	Overall (*n* = 3054)	HFrEF (*n* = 1720)	HFmrEF (*n* = 625)	HFpEF (*n* = 709)	*p*‐value
Age, years	69.2 ± 12.3	68.1 ± 11.9	68.0 ± 12.6	73.2 ± 12.2	<0.001
Female sex	756 (24.8)	323 (18.8)	144 (23.0)	289 (40.8)	<0.001
Caucasian	2925 (97.8)	1652 (97.6)	597 (98.2)	676 (97.8)	0.675
Italian region					0.228
North	1803 (59.0)	1006 (58.5)	355 (56.8)	442 (62.3)	
Centre	904 (29.6)	523 (30.4)	190 (30.4)	191 (26.9)	
South	347 (11.4)	191 (11.1)	80 (12.8)	76 (10.7)	
NYHA class					0.002
I/II	2486 (81.5)	1371 (79.8)	543 (86.9)	572 (80.7)	
III	546 (17.9)	333 (19.4)	80 (12.8)	133 (18.8)	
IV	20 (0.7)	14 (0.8)	2 (0.3)	4 (0.6)	
Outpatients	2514 (82.3)	1383 (80.4)	542 (86.7)	589 (83.1)	0.002
Baseline LVEF, %	40.0 ± 11.7	31.6 ± 6.5	44.8 ± 2.1	56.2 ± 5.1	<0.001
Current smoking	357 (11.8)	206 (12.0)	81 (13.0)	70 (10.0)	0.193
Clinical history					
*De novo* HF	471 (15.5)	250 (14.6)	82 (13.1)	139 (19.7)	0.001
Type 2 diabetes mellitus	924 (30.3)	559 (32.5)	163 (26.1)	202 (28.5)	0.006
Ischaemic CMP	1419 (46.7)	971 (56.7)	243 (39.2)	205 (29.0)	<0.001
Hypertension	1973 (64.8)	1047 (61.1)	399 (63.9)	527 (74.3)	<0.001
Atrial fibrillation	1281 (42.1)	686 (40.0)	238 (38.2)	357 (50.4)	<0.001
Chronic kidney disease	1130 (37.3)	672 (39.4)	185 (29.9)	273 (38.7)	<0.001
Dyslipidaemia	1983 (65.2)	1140 (66.7)	397 (63.6)	446 (63.0)	0.135
Dementia	34 (1.1)	15 (0.9)	7 (1.1)	12 (1.7)	0.216
History of ventricular arrhythmias or ICD shock	496 (16.3)	387 (22.6)	77 (12.4)	32 (4.5)	<0.001
ICD or CRT‐D	1345 (44.2)	1049 (61.1)	188 (30.1)	108 (15.4)	<0.001
Prior hospitalization in the last 12 months	645 (21.2)	413 (24.1)	89 (14.3)	143 (20.3)	<0.001
Vital signs and laboratory findings					
Heart rate, bpm	68.5 ± 12.9	68.6 ± 12.6	67.0 ± 11.9	69.4 ± 14.2	0.002
Systolic blood pressure, mmHg	120.8 ± 18.4	117.1 ± 17.3	122.9 ± 18.3	127.9 ± 18.7	<0.001
BMI, kg/m^2^	26.6 ± 5.1	26.5 ± 5.1	26.7 ± 4.9	26.7 ± 5.4	0.657
Overweight/obese	1540 (57.8)	877 (57.6)	323 (59.0)	340 (57.2)	0.796
Haemoglobin, g/dl	13.8 (12.4–15.1)	14.0 (12.6–15.3)	13.9 (12.4–15.1)	13.2 (12.0–14.6)	<0.001
eGFR, ml/min/1.73 m^2^					
<30	239 (7.8)	137 (8.0)	38 (6.1)	64 (9.0)	<0.001
30–60	1055 (34.6)	597 (34.7)	180 (28.8)	278 (39.2)	<0.001
>60	1759 (57.6)	986 (57.3)	406 (65.1)	367 (51.8)	<0.001
NT‐proBNP, pg/ml	922.0 (341.5–2494.5)	1207.0 (469.0–3067.0)	527.0 (207.8–1465.5)	784.0 (270.0–2228.5)	<0.001
K^+^, mmol/L	4.4 (4.1–4.8)	4.4 (4.1–4.8)	4.5 (4.2–4.9)	4.4 (4.1–4.7)	0.379

Data are expressed as *n* (%), mean ± standard deviation, or median (interquartile range).

BMI, body mass index; CMP, cardiomyopathy; CRT‐D, cardiac resynchronization therapy‐defibrillator; eGFR, estimated glomerular filtration rate; HF, heart failure; HFmrEF, heart failure with mildly reduced ejection fraction; HFpEF, heart failure with preserved ejection fraction; HFrEF, heart failure with reduced ejection fraction; ICD, implantable cardioverter‐defibrillator; LVEF, left ventricular ejection fraction; NT‐proBNP, N‐terminal pro‐B‐type natriuretic peptide; NYHA, New York Heart Association.

### Baseline medical therapy

**Figure 1:**
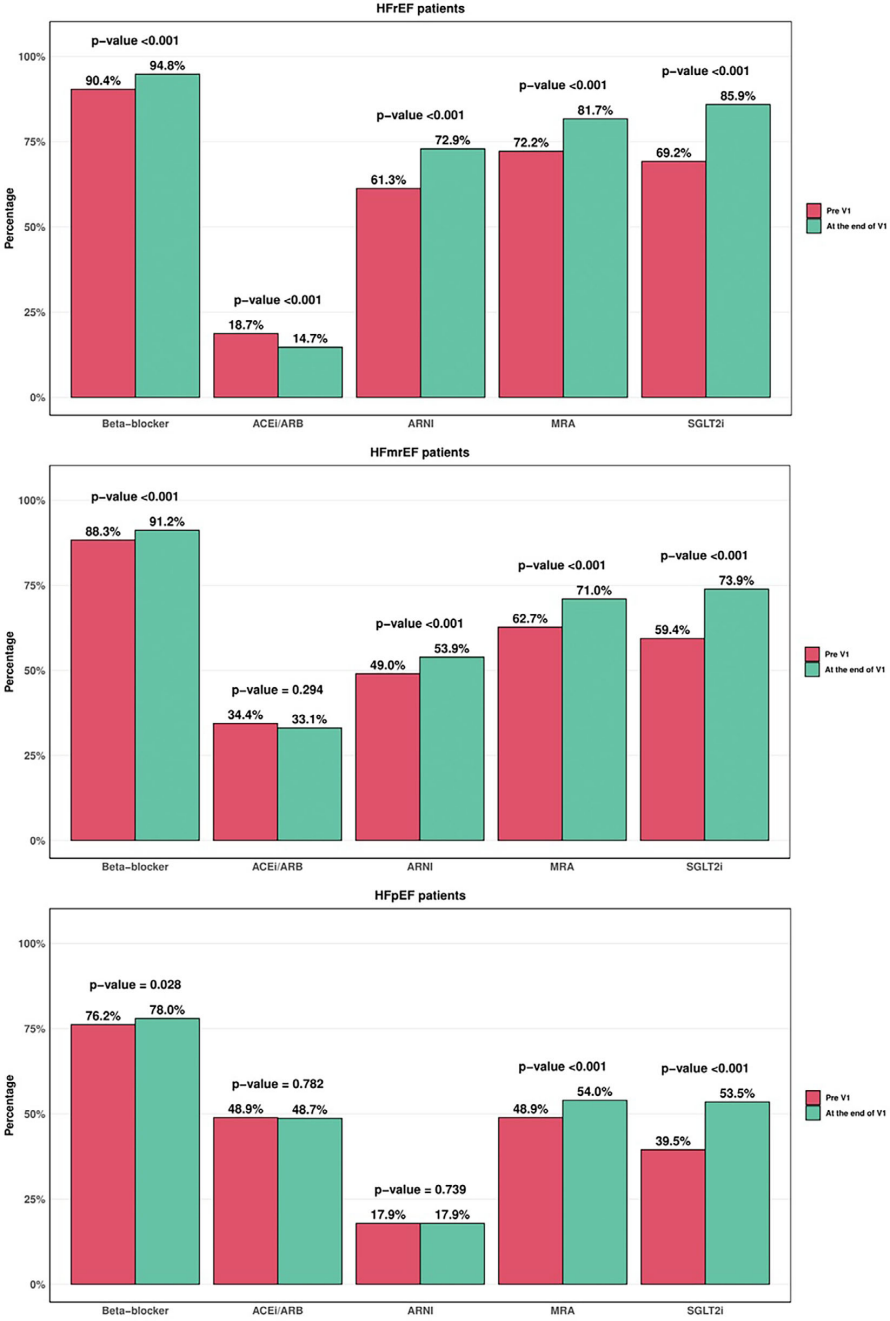
Baseline medical therapy and changes after V1 across the heart failure groups. ACEi, angiotensin‐converting enzyme inhibitor; ARB, angiotensin II receptor blocker; ARNI, angiotensin receptor–neprilysin inhibitor; HFmrEF, heart failure with mildly reduced ejection fraction; HFpEF, heart failure with preserved ejection fraction; HFrEF, heart failure with reduced ejection fraction; MRA, mineralocorticoid receptor antagonist; SGLT2i, sodium–glucose co‐transporter 2 inhibitor.

**Figure 2:**
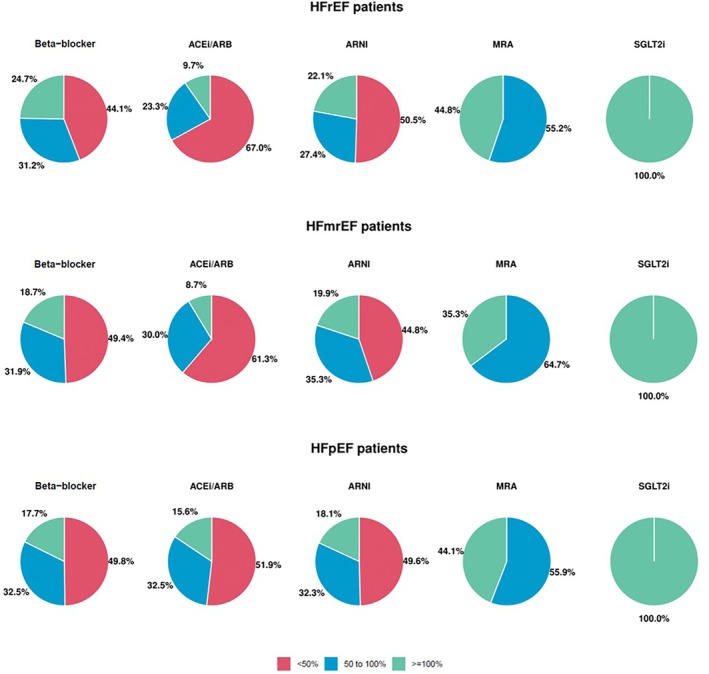
Proportion of patients with available dosage data treated with <50% target dose, 50% to <100% target dose, or ≥100% target dose. ACEi, angiotensin‐converting enzyme inhibitor; ARB, angiotensin II receptor blocker; ARNI, angiotensin receptor–neprilysin inhibitor; HFmrEF, heart failure with mildly reduced ejection fraction; HFpEF, heart failure with preserved ejection fraction; HFrEF, heart failure with reduced ejection fraction; MRA, mineralocorticoid receptor antagonist; SGLT2i, sodium–glucose co‐transporter 2 inhibitor.

**Figure 3:**
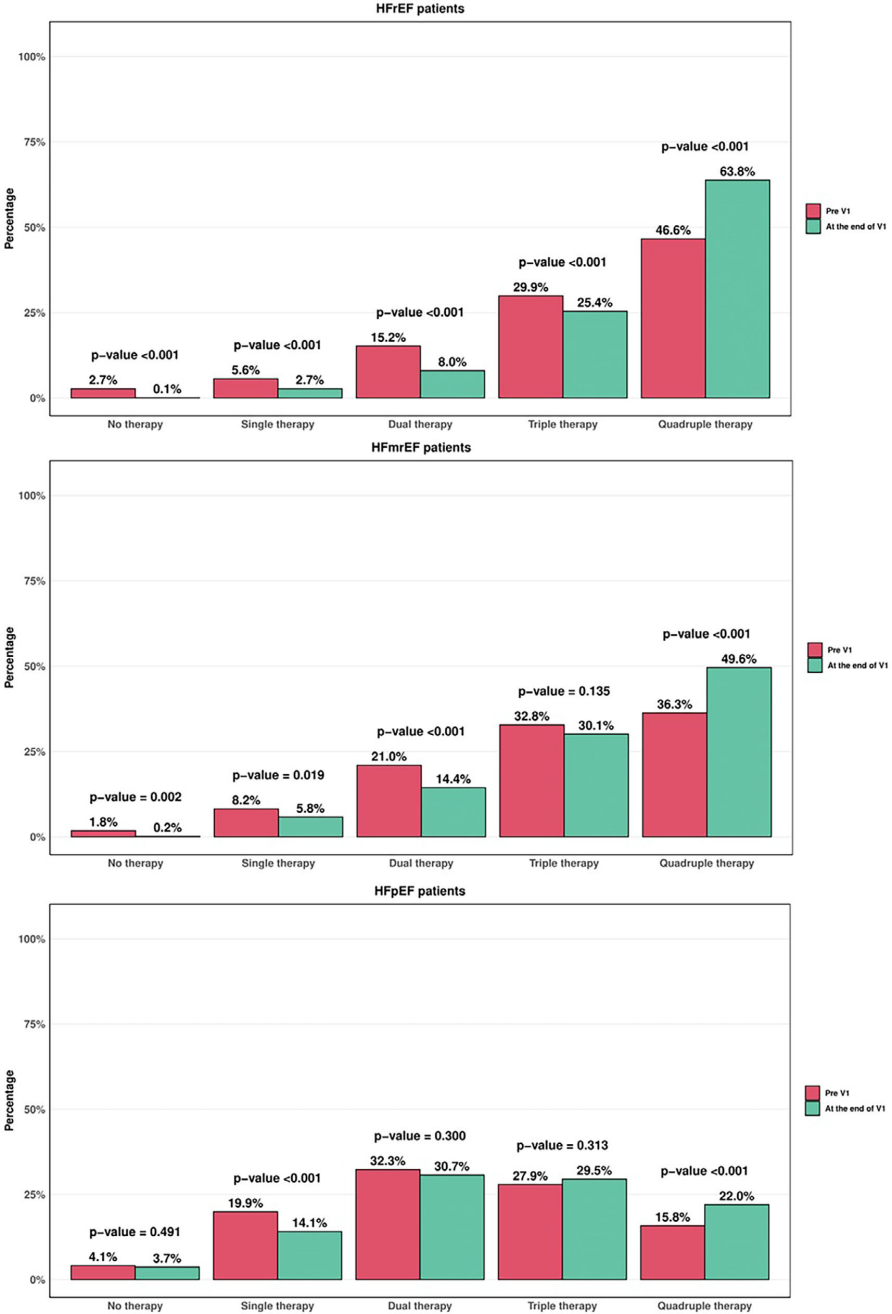
Baseline number of medications across the left ventricular ejection fraction spectrum and changes after V1. HFmrEF, heart failure with mildly reduced ejection fraction; HFpEF, heart failure with preserved ejection fraction; HFrEF, heart failure with reduced ejection fraction.

**Figure 4:**
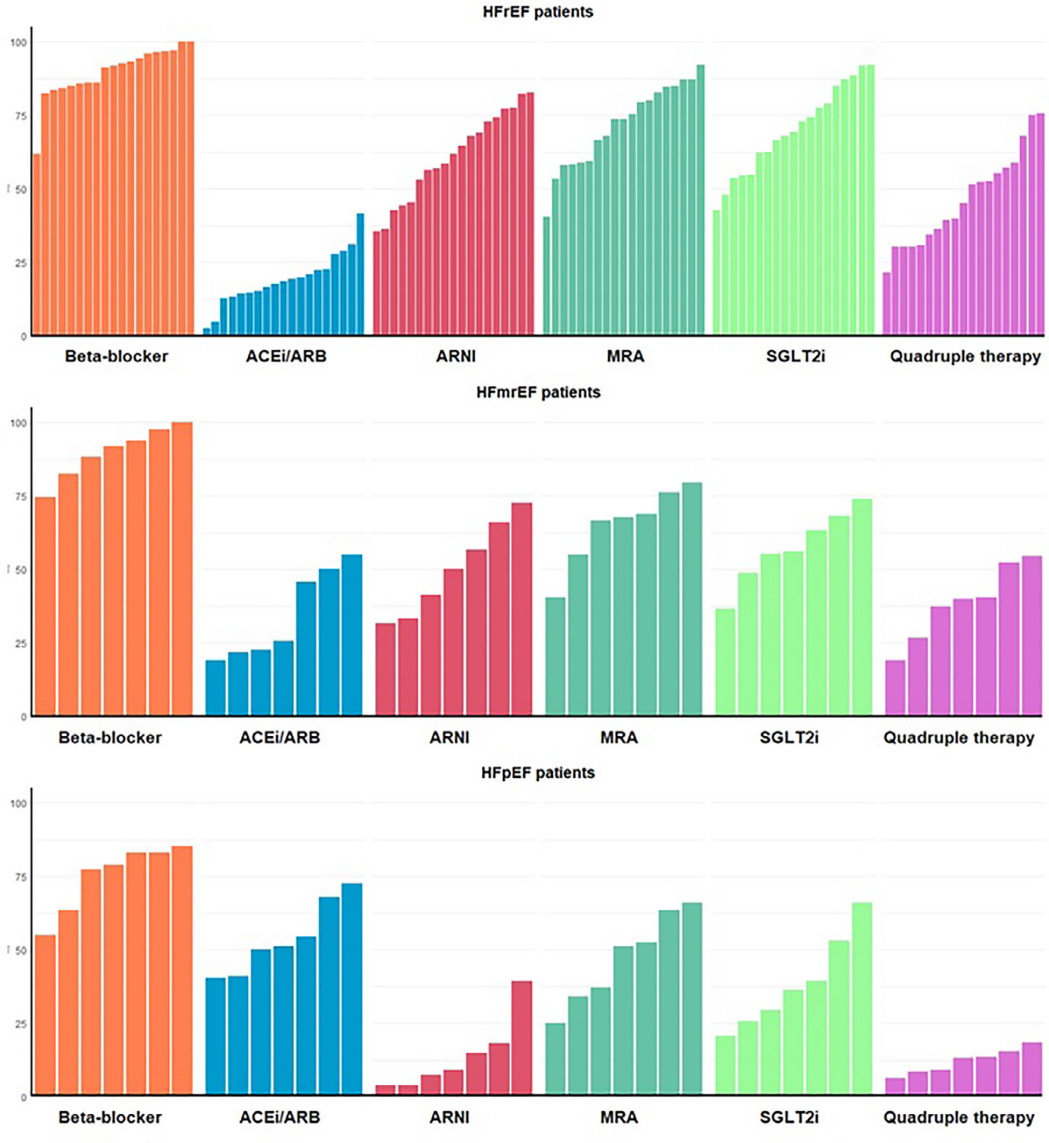
Differences in baseline medical therapy per participating sites. ACEi, angiotensin‐converting enzyme inhibitor; ARB, angiotensin II receptor blocker; ARNI, angiotensin receptor–neprilysin inhibitor; HFmrEF, heart failure with mildly reduced ejection fraction; HFpEF, heart failure with preserved ejection fraction; HFrEF, heart failure with reduced ejection fraction; MRA, mineralocorticoid receptor antagonist; SGLT2i, sodium–glucose co‐transporter 2 inhibitor.

In the HFrEF group, 1555 (90.4%) patients, 322 (18.7%), 1055 (61.3%), 1242 (72.2%) and 1191 (69.2%) were treated with beta‐blockers, angiotensin‐converting enzyme inhibitors (ACEi)/angiotensin II receptor blockers (ARB), ARNI, MRA and SGLT2i, respectively (*Graphical Abstract*, *Figure* *1*, *Table* *2*). Overall, 1377 (80%) patients were treated with ACEi/ARB/ARNI. The percentage of patients with a contraindication was low for all classes of drugs: 2.6% for beta‐blockers, 9.8% for ACEi/ARB, 15.7% for ARNI, 9.4% for MRA, and 6.2% for SGLT2i (*Graphical Abstract*, *Table* *3* and online supplementary *Figure* *S3*). Among patients receiving medications, 55.9%, 33.0%, 49.5%, 100% and 100%, respectively, achieved ≥50% of the target dose of beta‐blockers, ACEi/ARB, ARNI, MRA and SGLT2i (*Figure* *2*). Among patients receiving beta‐blockers, those receiving higher doses tended to be younger, obese, less likely to being hospitalized in the last 12 months, with a more frequent history of ventricular arrhythmias and lower values of NT‐proBNP (online supplementary *Table* *S1*). Among patients receiving MRA, those receiving higher doses tended to be younger, with lower LVEF and had been more frequently hospitalized in the last 12 months (online supplementary *Table* *S2*). Among patients receiving ARNI, those receiving higher doses were younger, more likely to be male, in the outpatient setting and with more preserved kidney function and lower values of NT‐proBNP (online supplementary *Table* *S3*). Overall, 46.6% of patients received quadruple therapy, 29.9% triple therapy, 15.2% double therapy, 5.6% single therapy and 2.7% of patients were without any therapy (*Table* *4* and *Figure* *3*). Patients receiving quadruple medical therapy were younger, more likely to be enrolled in Northern regions, with a lower burden of comorbidities and lower values of NT‐proBNP (online supplementary *Table* *S4*). Only 2.7% of patients were simultaneously treated with target dose of beta‐blockers, ARNI, MRA and SGLT2i. There were substantial differences between sites regarding medical therapy prescription. This was notable for all classes of drugs and for quadruple GDMT prescription, which varied from 25% to 75% across different sites (*Figure* *4*).

**Table 2:** Baseline medical therapy according to left ventricular ejection fraction categories

	Overall (*n* = 3054)	HFrEF (*n* = 1720)	HFmrEF (*n* = 625)	HFpEF (*n* = 709)	*p*‐value
Guideline‐directed medical therapy					
Beta‐blocker	2647 (86.7)	1555 (90.4)	552 (88.3)	540 (76.2)	<0.001
≥100% target dose	578 (22.0)	382 (24.7)	102 (18.7)	94 (17.7)	<0.001
ACEi/ARB	884 (28.9)	322 (18.7)	215 (34.4)	347 (48.9)	<0.001
≥100% target dose	99 (11.8)	30 (9.7)	18 (8.7)	51 (15.6)	0.020
ARNI	1488 (48.7)	1055 (61.3)	306 (49.0)	127 (17.9)	<0.001
≥100% target dose	317 (21.3)	233 (22.1)	61 (19.9)	23 (18.1)	0.473
ACEi/ARB/ARNI	2372 (77.7)	1377 (80.0)	521 (83.4)	474 (66.9)	<0.001
≥100% target dose	416 (17.5)	263 (19.1)	79 (15.2)	74 (15.6)	0.068
MRA	1981 (64.9)	1242 (72.2)	392 (62.7)	347 (48.9)	<0.001
≥100% target dose	847 (42.8)	556 (44.8)	138 (35.3)	153 (44.1)	0.004
SGLT2i	1842 (60.3)	1191 (69.2)	371 (59.4)	280 (39.5)	<0.001
Other medications					
Furosemide	1834 (60.1)	1120 (65.1)	317 (50.7)	397 (56.0)	<0.001
Daily dose, mg	25 (25–75)	50 (25–75)	25 (25–50)	40 (25–75)	0.001
Other diuretics	167 (5.5)	81 (4.7)	27 (4.3)	59 (8.3)	0.001
Vericiguat	86 (2.8)	74 (4.3)	10 (1.6)	2 (0.3)	<0.001
≥100% target dose	37 (43.0)	34 (46.0)	1 (10.0)	2 (100)	0.017
Ivabradine	146 (4.8)	96 (5.6)	30 (4.8)	20 (2.8)	0.015
Calcium channel blockers	304 (10.0)	113 (6.6)	50 (8.0)	141 (19.9)	<0.001
Digoxin/digitalis glycoside	133 (4.4)	75 (4.4)	28 (4.5)	30 (4.2)	0.975
Nitrates	131 (4.3)	83 (4.8)	22 (3.5)	26 (3.7)	0.261
Amiodarone	576 (18.9)	392 (22.8)	100 (16.0)	84 (11.9)	<0.001
Aspirin	1058 (34.7)	664 (38.7)	204 (32.6)	190 (27.0)	<0.001
Anticoagulation	1426 (46.7)	819 (47.6)	249 (39.9)	358 (50.6)	<0.001
Statin	2065 (67.7)	1212 (70.5)	423 (67.9)	430 (60.9)	<0.001

Data are expressed as *n* (%), median (interquartile range).

ACEi, angiotensin‐converting enzyme inhibitor; ARB, angiotensin II receptor blocker; ARNI, angiotensin receptor–neprilysin inhibitor; HFmrEF, heart failure with mildly reduced ejection fraction; HFpEF, heart failure with preserved ejection fraction; HFrEF, heart failure with reduced ejection fraction; MRA, mineralocorticoid receptor antagonist; SGLT2i, sodium–glucose co‐transporter 2 Inhibitor.

**Table 3:** Reasons for not receiving medical therapy

	HFrEF (*n* = 1720)					HFmrEF (*n* = 625)	HFpEF (*n* = 709)
	Beta‐blockers	ACEi/ARB	ARNI	MRA	SGLT2i	SGLT2i	SGLT2i
Not treated, contraindications, side effects, intolerance, *n* (%)	45 (2.6)	168 (9.8)	270 (15.7)	161 (9.4)	106 (6.2)	61 (9.7)	77 (10.9)
Allergic reaction	2 (4.4)	4 (2.4)	4 (1.5)	2 (1.2)	5 (4.7)	2 (3.3)	1 (1.3)
Bradycardia/AV block	31 (68.9)	–	–	–	–	–	–
Hypotension	11 (24.4)	101 (60.1)	167 (61.9)	29 (18.0)	10 (9.4)	16 (26.2)	20 (26.0)
Gynecomastia	–	–	–	5 (3.1)	–	–	–
Urinary tract infection/symptoms	–	–	–	–	45 (42.5)	22 (36.1)	29 (37.7)
Pulmonary disease	1 (2.2)	–	–	–	–	–	–
Chronic kidney disease	–	50 (29.8)	76 (28.1)	56 (34.8)	37 (34.9)	12 (19.7)	14 (18.2)
Hyperkalaemia	–	3 (1.8)	5 (1.9)	64 (39.8)	–	3 (4.9)	4 (5.2)
Previous creatinine increase	–	10 (6.0)	18 (6.7)	5 (3.1)	9 (8.5)	6 (9.8)	9 (11.7)
Not treated, other reasons, *n* (%)	120 (7.0)	1230 (71.5)^a^	395 (23.0)^b^	317 (18.4)	423 (24.6)	193 (30.9)	352 (49.6)
Patient preference	2 (1.7)	5 (0.4)	12 (3.0)	9 (2.8)	19 (4.5)	2 (1.0)	7 (2.0)
Other reasons	81 (67.5)	1121 (91.1)	147 (37.2)	108 (34.1)	142 (33.6)	69 (35.8)	154 (43.8)
Not known	37 (30.8)	104 (8.5)	236 (59.7)	200 (63.1)	262 (61.9)	122 (63.2)	191 (54.3)

AV, atrio‐ventricular; ACEi, angiotensin‐converting enzyme inhibitor; ARB, angiotensin II receptor blocker; ARNI, angiotensin receptor–neprilysin inhibitors; HFmrEF, heart failure with mildly reduced ejection fraction; HFpEF, heart failure with preserved ejection fraction; HFrEF, heart failure with reduced ejection fraction; MRA, mineralocorticoid receptor antagonist; SGLT2i, sodium–glucose co‐transporter‐2 inhibitor.

^a^ Of these, 85.7% were taking ARNI.

^b^ Of these, 56% were taking ACEi/ARB.

**Table 4:** Baseline medical therapy according to the number of medications across the left ventricular ejection fraction spectrum

	Overall (*n* = 3054)	HFrEF (*n* = 1720)	HFmrEF (*n* = 625)	HFpEF (*n* = 709)
No therapy	87 (2.9)	47 (2.7)	11 (1.8)	29 (4.1)
Single therapy	289 (9.5)	97 (5.6)	51 (8.2)	141 (19.9)
Beta‐blocker	144 (49.8)	54 (55.7)	24 (47.1)	66 (46.8)
MRA	33 (11.4)	6 (6.2)	8 (15.7)	19 (13.5)
ACEi/ARB/ARNI	88 (30.5)	27 (27.8)	17 (33.3)	44 (31.2)
SGLT2i	24 (8.3)	10 (10.3)	2 (3.9)	12 (8.5)
Dual therapy	621 (20.3)	261 (15.2)	131 (21.0)	229 (32.3)
Beta‐blocker + MRA	132 (21.3)	67 (25.7)	20 (15.3)	45 (19.7)
Beta‐blocker + ACEi/ARB/ARNI	308 (49.6)	123 (47.1)	72 (55.0)	113 (49.3)
Beta‐blocker + SGLT2i	64 (10.3)	29 (11.1)	13 (9.9)	22 (9.6)
MRA + ACEi/ARB/ARNI	43 (6.9)	14 (5.4)	10 (7.6)	19 (8.3)
MRA + SGLT2i	32 (5.2)	15 (5.8)	4 (3.1)	13 (5.7)
ACEi/ARB/ARNI + SGLT2i	42 (6.8)	13 (5.0)	12 (9.2)	17 (7.4)
Triple therapy	917 (30.0)	514 (29.9)	205 (32.8)	198 (27.9)
Beta‐blocker + MRA + ACEi/ARB/ARNI	377 (41.1)	191 (37.2)	92 (44.9)	94 (47.5)
Beta‐blocker + MRA + SGLT2i	166 (18.1)	115 (22.4)	22 (10.7)	29 (14.7)
Beta‐blocker + ACEi/ARB/ARNI + SGLT2i	316 (34.5)	175 (34.1)	82 (40.0)	59 (29.8)
MRA + ACEi/ARB/ARNI + SGLT2i	58 (6.3)	33 (6.4)	9 (4.4)	16 (8.1)
Quadruple therapy	1140 (37.3)	801 (46.6)	227 (36.3)	112 (15.8)

Data are presented as *n* (%).

ACEi, angiotensin‐converting enzyme inhibitor; ARB, angiotensin II receptor blocker; ARNI, angiotensin receptor–neprilysin inhibitors; HFmrEF, heart failure with mildly reduced ejection fraction; HFpEF, heart failure with preserved ejection fraction; HFrEF, heart failure with reduced ejection fraction; MRA, mineralocorticoid receptor antagonist; SGLT2i, sodium–glucose co‐transporter‐2 inhibitor.

Among the HFmrEF group, 552 (88.3%) patients were on beta‐blockers, 215 (34.4%) patients were on ACEi/ARB, 306 (49.0%) patients were on ARNI (a total of 521 [83%] on ACEi/ARB/ARNI), 392 (62.7%) were on MRA, and 371 (59.4%) were on SGLT2i (*Graphical Abstract*, *Figure* *1*). Of these, 50.6%, 38.7%, 55.5%, 100% and 100% received ≥50% of the target dose, respectively (*Figure* *2*). The percentage of contraindication for SGLT2i was 9.7% (*Graphical Abstract*). Of note 36.3% were prescribed quadruple therapy (*Table* *4* and *Figure* *3*).

In the HFpEF group, the prescription rate for SGLT2i was 39% and 10.9% had contraindication for treatment (*Graphical Abstract*, *Figure* *1*). Overall, 76.2% were on beta‐blockers, 48.9% on ACEi/ARB, 17.9% on ARNI (a total of 66.8% on ACEi/ARB/ARNI), 48.9% on MRA.

### Changes in medical therapy after the first visit

Among HFrEF patients, 5.2% initiated beta‐blockers, 3.6% initiated ACEi/ARB, 12.9% initiated ARNI, 11.2% initiated MRA, and 17.2% initiated SGLT2i after V1 (*Figure* *1*). Among those who already received medications but not on target dose, 7.3% increased the dose of beta‐blocker, 3.4% of ACEi/ARB, 15.3% of ARNI, 10.0% of MRA. The rate of discontinuation was 0.8%, 7.6%, 1.3%, 1.7%, 0.5% for beta‐blockers, ACEi/ARB, ARNI, MRA and SGLT2i, respectively, and 37.3% switched from ACEi/ARB to ARNI. Overall, 18.5% initiated quadruple medical therapy at the end of V1 (*Figure* *3*) and 6.6% were simultaneously treated with target doses of beta‐blockers, ARNI, MRA and SGLT2i.

Among HFmrEF patients, 3.7%, 4.0%, 5.6%, 9.8%, 15.4%, initiated beta‐blockers, ACEi/ARB, ARNI, MRA and SGLT2i, respectively (*Figure* *1*). Discontinuation rate was 0.8%, 5.3%, 0.6%, 1.4%, 0.8%, for beta‐blockers, ACEi/ARB, ARNI, MRA and SGLT2i, respectively.

In the HFpEF group, 15.1% initiated SGLT2i at the end of V1 and 1.0% discontinued this treatment. Of note, initiation of beta‐blockers, ACEi/ARB, ARNI, and MRA was 3.4%, 3.5%, 0.7% and 7.8%, respectively (*Figure* *1*).

## Discussion

In this large contemporary national HF population enrolled in the OPTIPHARM‐HF registry, we found a significant gap in guideline‐recommended use and dosing of evidence‐based treatment across the spectrum of ejection fraction. Among HFrEF patients, despite the low prevalence of evident contraindication for each medication, use of recommended treatment was below 75% with the exception of beta‐blockers. Among patients receiving medications, beta‐blockers, ACEi/ARB/ARNI and MRA were mostly prescribed at sub‐target doses. Less than 50% of patients simultaneously received any dose of all four recommended medications. At the end of V1, most eligible patients did not receive target doses of medical therapy, and few patients had increased doses. We, however, observed a significant increase in prescription rates of beta‐blockers, MRA and ARNI, and 85% of patients were prescribed SGLT2i. Less than 60% of the population received quadruple therapy after the first visit. For patients with mildly reduced or preserved ejection fraction, baseline treatment with SGLT2i was less than 60% despite the low prevalence of contraindications and the indication in the ESC HF guideline update and less than 75% were prescribed SGLT2i at the end of first visit.

The current primary analysis of the OPTIPHARM‐HF registry provides a unique and timely contribution to the field by offering one of the most comprehensive contemporary assessments of GDMT implementation across the full spectrum of LVEF in a large, national HF population (*Graphical Abstract*). Unlike prior registries that have primarily focused on HFrEF or relied on administrative data with limited granularity, OPTIPHARM‐HF includes detailed, prospectively collected information on clinical characteristics, comorbidities, drug dosing, and titration strategies across HFrEF, HFmrEF, and HFpEF. In particular, this is one of the first studies to systematically evaluate the real‐world adoption of SGLT2i across ejection fraction phenotypes, including detailed data on initial prescription rates, contraindications, and changes following specialist assessment with data collection timelines including the time of approval of SGLT2i for the treatment of patients with HFpEF.^5^, ^6^

In previous reports, GDMT implementation in HFrEF varied from 70% to 90% for beta‐blockers, from 10% to 57% for ARNI, and from 30% to 75% for MRA. In OPTIPHARM‐HF, we observed a higher baseline medical therapy in comparison to previous large‐scale HF registries, such as CHECK‐HF, CHAMP‐HF, and ASIAN‐HF,^11^, ^12^, ^13^ which were conducted before the widespread adoption of newer therapies like SGLT2i and prior to recent guideline updates. In contrast, the most recent TITRATE‐HF and SwedeHF registries and the VICTOR trial,^14^, ^15^, ^16^ present a similar rate of baseline medical therapy to our population. However, prescribed doses were still significantly below target doses. Data on the implementation of SGLT2i are still scarce due to their recent introduction. In the SwedeHF registry, ∼50% of HFrEF patients received a SGLT2i while in TITRATE‐HF 65% were prescribed a SGLT2i, and 59% in the VICTOR trial. We found a slightly higher treatment rate with SGLT2i with a significant change in the prescription rate after V1 reaching 85% of the patients. The higher rate of SGLT2i implementation in TITRATE‐HF and OPTIPHARM‐HF likely reflects the timing of enrolment, capturing a more contemporary period after broader regulatory approvals and integration of SGLT2i into clinical algorithms and guideline updates as compared to the SwedeHF. As observed in the TITRATE‐HF where 44% were prescribed quadruple therapy, we found comparable data as less than 50% received all GDMT at the time of enrolment. This is in line with findings from the multinational EVOLUTION‐HF, which also demonstrated delayed initiation of novel drug classes.^17^ More recently, the IMPLEMENT‐HF showed a significant increase in the use of quadruple medical therapy reaching 44.8% among HFrEF patients discharged after a recent hospitalization for HF.^18^

OPTIPHARM‐HF represents one of the first registries assessing the prescription of medical therapy and particularly SGLT2i for HF patients with LVEF >40%. Recent data from the Get With The Guidelines‐Heart Failure registry showed an increase in prescription rate of SGLT2i at hospital discharge from 4.2% to 23.5% in the period 2021–2023.^19^ These data, largely derived from hospitalized U.S. populations, contrast with the broader outpatient and inpatient enrolment in OPTIPHARM‐HF and the structured HF‐focused evaluation conducted during study visits, likely contributing to the higher prescription rates observed in our cohort. In FINEARTS‐HF, only 13.6% of enrolled patients were treated with SGLT2i.^20^ Although in OPTIPHARM‐HF approximately 40% of HFmrEF and HFpEF patients were not prescribed a SGLT2i, a promising increase in prescription after V1 was observed. Ongoing follow‐up visits will better define the prescription rate of SGLT2i over time in this population.

Interestingly, we observed relatively high background treatment with beta‐blockers both in the HFmrEF and HFpEF groups. These data contrast with the lack of benefits of beta‐blockers in patients with HFpEF but are, however, in line with the results in FINEARTS‐HF (85%) and EMPEROR‐Preserved (86%).^20^, ^21^ Regarding ARNI and MRA, we observed a progressive decrease in their use from HFmrEF to HFpEF; however, the overall prevalence of these therapies remained higher compared to recent clinical trials enrolling patients with HFmrEF and HFpEF. Of note, data on medical therapy use and target dose in HFmrEF and HFpEF should be interpreted with caution, as this represents an analytic framework rather than evidence‐based guidance. Current guidelines do not provide specific drug‐ or dose‐related recommendations for these phenotypes beyond SGLT2i.

Taken together the results of OPTIPHARM‐HF emphasize the persistent unmet need for generalizable strategies to improve widespread use and dosing of GDMT,^7^, ^22^, ^23^ similarly to what is reported to device implantation recommended in HFrEF.^24^ Although all patients received care at specialized cardiology centres, a substantial gap in GDMT use persists across all HF phenotypes, highlighting the need for further efforts to enhance care quality. These gaps are not solely attributable to clinical contraindications, as most patients did not exhibit limiting reasons for not receiving medical therapy. Instead, they likely reflect a combination of therapeutic inertia, limited awareness or confidence in newer guideline updates, competing clinical priorities during time‐limited visits, and a lack of systematic care pathways. This issue is particularly relevant for patients with an LVEF >40%, for whom current adherence to recommended therapies remains low. In line with prior evidence, the underrepresentation of women in OPTIPHARM‐HF mirrors a broader pattern observed in both registries and clinical trials, with multiple barriers contributing to this imbalance. Structural and social factors, including delayed referral to specialty care, lower screening and enrolment in trials, and competing caregiving responsibilities, further exacerbate disparities. Women often present at an older age, with lower body weight, and more frequent adverse events such as hypotension or bradycardia, which may limit achievement of target GDMT doses.

The observed increase in prescription rates of key GDMT classes after V1 highlights the potential impact of even a single structured clinical visit in optimizing HF care. This suggests that a more structured and proactive model of HF management—including dedicated visits for medication review and titration—could be instrumental in addressing the persistent therapeutic gap. Although the study reflects real‐world clinical practice in Italy, the enrolment visit itself worked as an optimization encounter, revealing how much can be achieved with systematic and intentional care planning.^25^ To this regard, the long‐term design of the OPTIPHARM‐HF will help to assess sequencing, order and target dose of GDMT over time and the impact on clinical outcomes. This will allow for a better understanding of contemporary implementation barriers.

The OPTIPHARM‐HF registry has several limitations that merit consideration when interpreting the findings. Firstly, the national design of this registry potentially limits the external generalizability of our results as healthcare delivery, medical practice patterns, and patient populations may vary substantially in different geographic or international contexts. Furthermore, a significant proportion of patients were recruited from specialized centres, where adherence to GDMT tends to be higher compared to general clinical practice. This recruitment strategy may result in an overestimation of GDMT use, representing a potential selection bias. However, undertreatment and underdosing are likely to be even higher in non‐specialized centres. Additionally, the enrolled cohort was predominantly male and white, reflecting a demographic imbalance frequently observed in clinical registries and trials (online supplementary *Table* *S5*), thus limiting the applicability of our results to women and other racial or ethnic groups. This demographic limitation might mask clinically significant differences in therapeutic approaches, outcomes, or responses to therapy across more diverse populations. Another important consideration is that the case report forms utilized in the registry may not fully capture all clinical variables that could influence treatment implementation, such as socioeconomic status, educational background, healthcare accessibility, and lifestyle factors. Consequently, residual unmeasured variables might still influence the observed results. Finally, as the OPTIPHARM‐HF registry is still ongoing, the present analysis does not include clinical outcome data, which will be addressed in prespecified follow‐up analyses.^9^

The OPTIPHARM‐HF registry offers comprehensive data on medical therapy across the full spectrum of LVEF in a contemporary HF population by capturing detailed real‐world patterns of GDMT use—including drug class initiation, titration, and dosing—with a particular focus on the uptake of SGLT2i across ejection fraction phenotypes. Although the observed prescription rates for recommended treatment were higher compared to those observed in previous reports, persistent gaps remain in both the initiation and implementation of foundational therapies across the LVEF spectrum. These identified gaps in care underscore a critical unmet need in clinical practice with an urgent call for evidence‐based interventions and systematic strategies aimed at enhancing the care of HF patients.

## Supporting information


**Appendix S1.** Supporting Information.

## Appendix

Marianna Adamo^1^, Marilisa Ambrosio^26^, Irene Baroni^12,13^, Paolo Biagioli^20^, Valeria Borelli^15^, Natale Brunetti^23^, Margherita Calcagnino^15^, Giuseppe Caminiti^38,39^, Francesco Canonico^24^, Angelo Caporotondi^29^, Velia Cassano^34^, Ali Chraim^17^, Valentina Codazzi^1^, Giuliana Cimino^22^, Francesco Clemenza^22^, Giuditta Cuccuru^16^, Giulia De Lio^16^, Giandomenico Disabato^12,13^, Marcello Divino^9^, Salvatore D'Isa^26^, Roberta Famiani^15^, Enrica Fede^20^, Letizia Fiòorentino^1^, Alberto Foa'^32^, Paolo Fornaro^1^, Noemi Furlotti^17^, Nicolò Gallingani^32^, Alberto Genova^10^, Stefano Ghio^30^, Federico Gobbi^20^, Daniela Grassano^20^, Lucia Guerisoli^8^, Manuela Iseppi^19^, Silvia Laterza^32^, Simona Leone^22^, Ferdinando Loiacono^15^, Valeria Luberto^17^, Rossella Manai^16^, Niccolò Manetti^34^, Lina Manzi^19^, Fabio Marsico^36^, Francesco Maruca^9^, Gabriele Masini^25^, Irene Mattavelli^5^, Ciro Mauro^36^, Alberto Mazzoni^1^, Marta Mazzotta^1^, Pierre Meynet^16^, Valentina Morsella^38,39^, Giulia Nemola^17^, Martina Niccolai^10^, Savina Nodari^1^, Matteo Pagnesi^1^, Gianpaolo Palmieri^23^, Alberto Panza^9^, Francesca Parisi^22^, Carlo Alberto Pastura^9^, Anna Lisa Picerni^10^, Riccardo Pilia^16^, Ilaria Pirrone^17^, Nicola R. Pugliese^40^, Claudio Puteo^23^, Federica Ramani^19^, Gloria Santangelo^15^, Laura Scelsi^30^, Edoardo Sciatti^26^, Lorenza Sforzini^17^, Giulia Spoto^30^, Davide Stolfo^28^, Roberto Tarantini^30^, Maria Cristina Tavera^34^, Floriana Tortomasi^22^, Eduardo Valente^16^, Giuseppe Varricchione^38,39^, Enrico Vizzardi^1^, Greta Zagni^32^, Mattia Zampieri^27^
