# Supplementary material for: Contemporary medical therapy for heart failure across the ejection fraction spectrum: The OPTIPHARM‐HF registry
Source: Eur J Heart Fail. 2025 Dec 17;27(12):2691–704. doi: 10.1002/ejhf.70074 (PMC12803666; doi:10.1002/ejhf.70074)
Supplement: Supplementary file 1 — Appendix S1. Supporting Information. [file EJHF-27-2691-s001.docx]

**Appendices - SUPPLEMENTARY MATERIALS**

**Supplemental Figure 1**. Rate of recruitment over the enrollment period


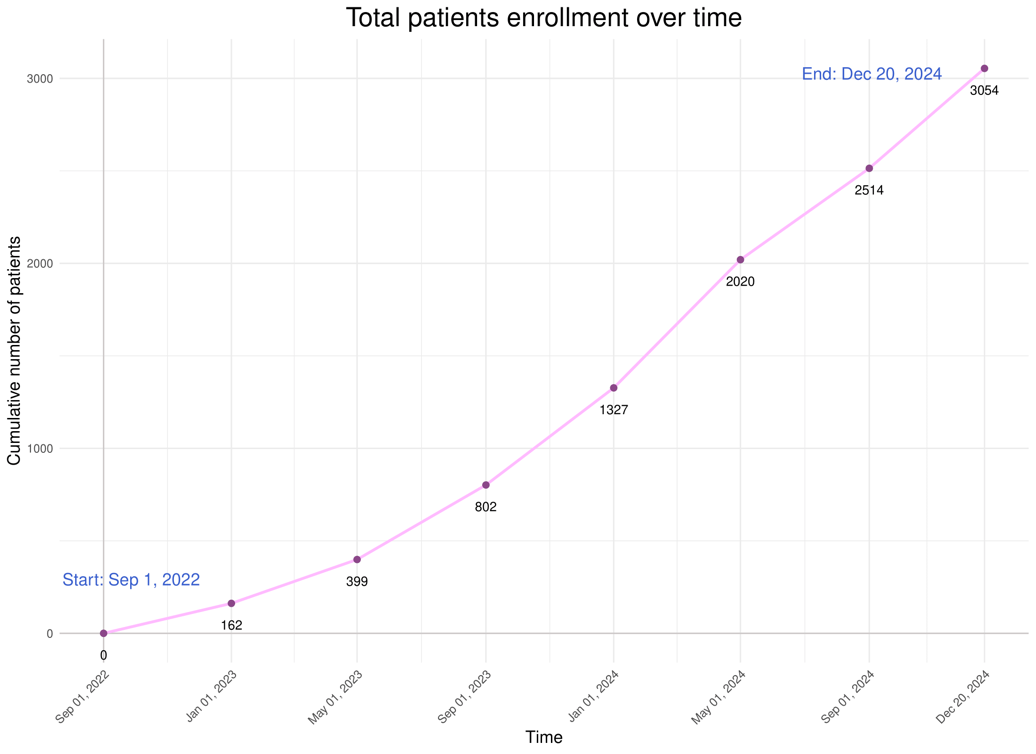


**Supplemental Figure 2.** Patients enrlolled per center in the OPTIPHARM-HF


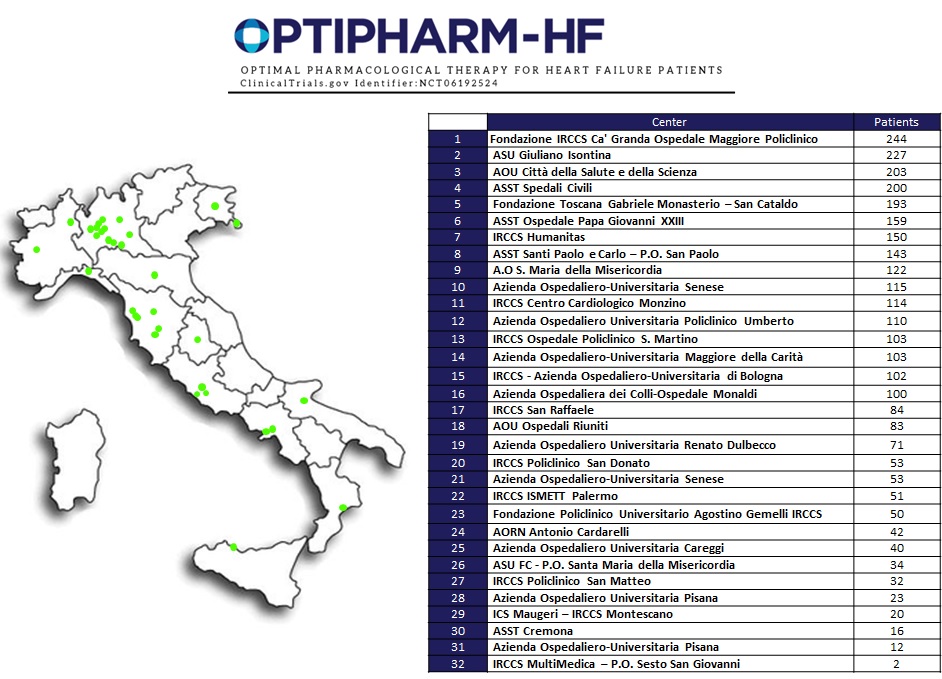


**
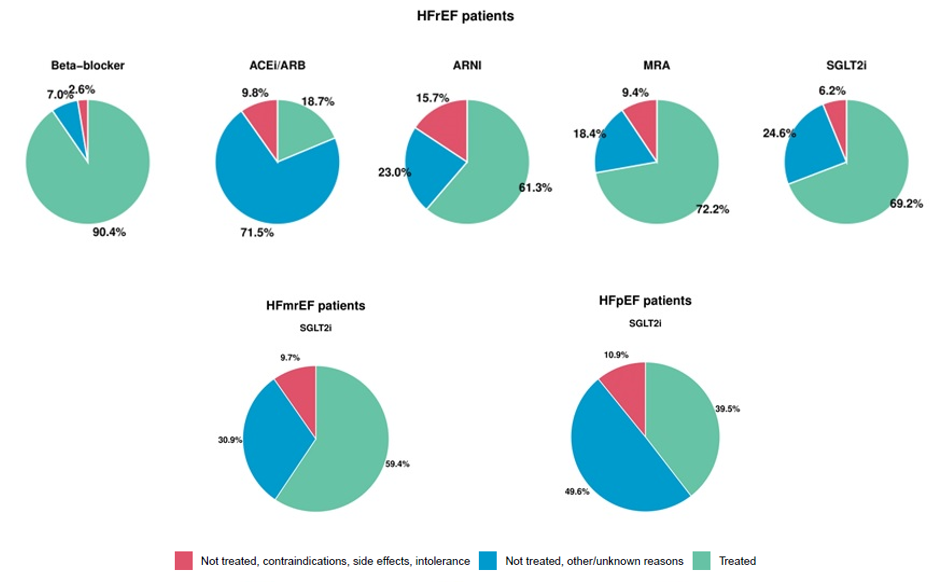
**

**Supplemental Figure 3** Proportion of the study population with a contraindication, treated with any dose, and without a contraindication but not treated.

**Supplemental Table 1.** Baseline HFrEF patient characteristics by dose of Beta-blocker

|  | **<50% target dose**  **(N=681)** | **50% to <100% target dose (N=483)** | **≥100% target dose (N=382)** | ***p-value*** |
| --- | --- | --- | --- | --- |
| Age, years | 69.2 ± 12.2 | 67.5 ± 11.7 | 66.5 ± 11.0 | *0.001* |
| Female | 141 (20.7) | 82 (17.0) | 63 (16.5) | *0.138* |
| Caucasian | 658 (98.1) | 464 (97.3) | 362 (96.8) | *0.418* |
| Italian region |  |  |  |  |
| - North | 418 (61.4) | 283 (58.6) | 200 (52.4) | *0.055* |
| - Centre | 193 (28.3) | 138 (28.6) | 129 (33.8) |  |
| - South | 70 (10.3) | 62 (12.8) | 53 (13.9) |  |
| NYHA |  |  |  |  |
| - I/II | 536 (78.8) | 378 (78.4) | 322 (84.3) | *0.037* |
| - III | 142 (20.9) | 98 (20.3) | 59 (15.4) |  |
| - IV | 2 (0.3) | 6 (1.2) | 1 (0.3) |  |
| Outpatients | 542 (79.6) | 395 (81.8) | 341 (89.3) | *<0.001* |
| Baseline LVEF, % | 31.6 ± 6.8 | 31.5 ± 6.4 | 31.4 ± 6.1 | *0.885* |
| Current smoking | 70 (10.3) | 63 (13.2) | 44 (11.6) | *0.321* |
| **Clinical history** |  |  |  |  |
| *De Novo* HF | 95 (14.0) | 55 (11.4) | 26 (6.8) | *0.002* |
| Type 2 diabetes mellitus | 216 (31.7) | 155 (32.2) | 140 (36.6) | *0.228* |
| Coronary artery disease | 391 (57.6) | 282 (58.4) | 195 (51.3) | *0.076* |
| Hypertension | 410 (60.6) | 290 (60.3) | 241 (63.3) | *0.619* |
| Atrial fibrillation | 246 (36.2) | 197 (41.0) | 178 (46.8) | *0.003* |
| Chronic kidney disease | 267 (39.3) | 196 (41.4) | 157 (41.6) | *0.678* |
| Dyslipidemia | 451 (66.5) | 328 (68.9) | 255 (67.1) | *0.688* |
| Dementia | 8 (1.2) | 5 (1.0) | 2 (0.5) | *0.641* |
| History of ventricular arrhythmias or ICD shock | 115 (16.9) | 116 (24.1) | 123 (32.2) | *<0.001* |
| ICD or CRT-D | 362 (53.2) | 329 (68.3) | 300 (78.7) | *<0.001* |
| Prior hospitalization in the last 12 months | 201 (29.6) | 123 (25.6) | 66 (17.3) | *<0.001* |
| **Vital Signs and laboratory findings** |  |  |  |  |
| Heart rate, beats/min | 67.8 ± 11.6 | 68.1 ± 12.9 | 70.1 ± 13.3 | *0.009* |
| Systolic blood pressure, mmHg | 116.5 ± 17.9 | 116.3 ± 16.8 | 119.0 ± 16.9 | *0.042* |
| BMI, kg/m^2^ | 25.8 ± 4.6 | 26.8 ± 5.4 | 27.7 ± 5.2 | *<0.001* |
| Overweight/Obese | 311 (51.7) | 259 (61.1) | 226 (66.9) | *<0.001* |
| Hemoglobin, g/dl | 13.7 (12.3-15.1) | 14.0 (12.4-15.3) | 14.5 (13.2-15.5) | *<0.001* |
| eGFR, ml/min/1.73 m^2^ |  |  |  |  |
| - < 30 | 59 (8.7) | 39 (8.1) | 30 (7.9) | *0.972* |
| - 30-60 | 230 (33.8) | 166 (34.4) | 136 (35.6) |  |
| - > 60 | 392 (57.6) | 278 (57.6) | 216 (56.5) |  |
| NT-proBNP, pg/ml | 1382.0 (534.3-3439.0) | 1228.0 (504.0-3207.0) | 927.0 (362.0-2277.3) | *0.001* |
| K+, mmol/L | 4.5 (4.1-4.8) | 4.4 (4.0-4.7) | 4.5 (4.2-4.8) | *0.013* |

Results are expressed as n (%), mean ± standard deviation, or median [interquartile range]. Abbreviations: BMI = body mass index; CRT-D = cardiac resynchronization therapy-defibrillator; eGFR = estimated glomerular filtration rate; ICD = implantable cardioverter defibrillator; NT-proBNP = N-terminal pro b-type natriuretic peptide; NYHA = New York Heart Association;

|  | **50% to <100% target dose (N=685)** | **≥100% target dose (N=556)** | ***p-value*** |
| --- | --- | --- | --- |
| Age, years | 68.4 ± 11.6 | 64.8 ± 12.3 | *<0.001* |
| Female Sex | 140 (20.4) | 109 (19.6) | *0.769* |
| Caucasian | 656 (97.2) | 531 (97.1) | *1* |
| Region |  |  |  |
| - Northern Italy | 415 (60.6) | 298 (53.6) | *<0.001* |
| - Centre Italy | 213 (31.1) | 157 (28.2) |  |
| - Southern Italy | 57 (8.3) | 101 (18.2) |  |
| NYHA |  |  |  |
| - I/II | 560 (81.8) | 426 (76.8) | *0.086* |
| - III | 122 (17.8) | 125 (22.5) |  |
| - IV | 3 (0.4) | 4 (0.7) |  |
| Outpatients | 587 (85.7) | 464 (83.5) | *0.312* |
| Baseline LVEF, % | 31.8 ± 6.3 | 30.3 ± 7.0 | *<0.001* |
| Current smoking | 85 (12.5) | 48 (8.7) | *0.039* |
| **Clinical history** |  |  |  |
| *De Novo* HF | 64 (9.4) | 64 (11.6) | *0.246* |
| Type 2 diabetes mellitus | 218 (31.9) | 166 (29.9) | *0.483* |
| Coronary artery disease | 370 (54.2) | 296 (53.4) | *0.839* |
| Hypertension | 404 (59.3) | 321 (57.8) | *0.638* |
| Atrial fibrillation | 292 (42.8) | 222 (39.9) | *0.344* |
| Chronic kidney disease | 279 (41.2) | 198 (36.0) | *0.075* |
| Dyslipidemia | 446 (65.4) | 368 (66.5) | *0.716* |
| Dementia | 6 (0.9) | 4 (0.7) | *1* |
| History of ventricular arrhythmias or ICD shock | 168 (24.7) | 148 (26.6) | *0.474* |
| ICD or CRT-D | 454 (66.4) | 388 (69.9) | *0.206* |
| Prior hospitalization in the last 12 months | 160 (23.5) | 169 (30.5) | *0.007* |
| **Vital Signs and laboratory findings** |  |  |  |
| Heart rate, beats/min | 68.1 ± 12.6 | 68.8 ± 12.4 | *0.344* |
| Systolic blood pressure, mmHg | 116.7 ± 16.3 | 113.5 ± 16.3 | *0.001* |
| BMI, kg/m^2^ | 26.4 ± 4.9 | 26.8 ± 5.6 | *0.234* |
| Overweight/Obese | 350 (57.4) | 291 (58.9) | *0.652* |
| Hemoglobin, g/dl | 14.2 (12.9-15.5) | 14.0 (12.5-15.3) | *0.042* |
| eGFR, ml/min/1.73 m^2^ |  |  |  |
| - < 30 | 46 (6.7) | 33 (5.9) | *0.546* |
| - 30-60 | 242 (35.3) | 184 (33.1) |  |
| - > 60 | 397 (58.0) | 339 (61.0) |  |
| NT-proBNP, pg/ml | 1168.0 (443.5-2597.0) | 1214.0 (484.0-3308.5) | *0.197* |
| K+, mmol/L | 4.5 (4.2-4.9) | 4.4 (4.0-4.7) | *<0.001* |

**Supplemental Table 2.** Baseline HFrEF patient characteristics by dose of MRA.

Results are expressed as n (%), mean ± standard deviation, or median [interquartile range. Abbreviations: BMI = body mass index; CRT-D = cardiac resynchronization therapy-defibrillator; eGFR = estimated glomerular filtration rate; ICD = implantable cardioverter defibrillator; NT-proBNP = N-terminal pro b-type natriuretic peptide; NYHA = New York Heart Association;

**Supplemental Table 3.** Baseline HFrEF patient characteristics by dose of ARNI.

|  | **<50% target dose**  **(N=533)** | **50% to <100% target dose (N=289)** | **≥100% target dose (N=233)** | ***p-value*** |
| --- | --- | --- | --- | --- |
| Age, years | 67.6 ± 11.6 | 65.6 ± 11.5 | 63.7 ± 11.4 | *<0.001* |
| Female Sex | 116 (21.8) | 34 (11.8) | 21 (9.0) | *<0.001* |
| Caucasian | 509 (97.1) | 279 (97.2) | 222 (98.7) | *0.443* |
| Region |  |  |  |  |
| - Northern Italy | 289 (54.2) | 168 (58.1) | 132 (56.7) | *0.186* |
| - Centre Italy | 176 (33.0) | 74 (25.6) | 73 (31.3) |  |
| - Southern Italy | 68 (12.8) | 47 (16.3) | 28 (12.0) |  |
| NYHA |  |  |  |  |
| - I/II | 409 (76.7) | 245 (85.4) | 211 (90.6) | *<0.001* |
| - III | 120 (22.5) | 39 (13.6) | 22 (9.4) |  |
| - IV | 4 (0.8) | 3 (1.0) | 0 |  |
| Outpatients | 452 (84.8) | 252 (87.2) | 221 (94.8) | *<0.001* |
| Baseline LVEF, % | 31.3 ± 6.7 | 31.6 ± 6.1 | 31.6 ± 5.9 | *0.675* |
| Current smoking | 64 (12.1) | 37 (12.9) | 31 (13.3) | *0.883* |
| **Clinical history** |  |  |  |  |
| *De Novo* HF | 62 (11.7) | 22 (7.6) | 2 (0.9) | *<0.001* |
| Type 2 diabetes mellitus | 160 (30.1) | 93 (32.2) | 73 (31.3) | *0.814* |
| Coronary artery disease | 295 (55.7) | 156 (54.0) | 129 (55.6) | *0.889* |
| Hypertension | 315 (59.2) | 173 (60.3) | 134 (57.8) | *0.845* |
| Atrial fibrillation | 202 (38.0) | 118 (40.8) | 87 (37.3) | *0.66* |
| Chronic kidney disease | 216 (40.8) | 88 (30.8) | 84 (36.7) | *0.018* |
| Dyslipidemia | 357 (67.4) | 206 (71.3) | 161 (69.1) | *0.509* |
| Dementia | 5 (0.9) | 2 (0.7) | 1 (0.4) | *0.899* |
| History of ventricular arrhythmias or ICD shock | 127 (24.0) | 71 (24.6) | 69 (29.7) | *0.224* |
| ICD or CRT-D | 334 (62.7) | 211 (73.0) | 193 (83.5) | *<0.001* |
| Prior hospitalization in the last 12 months | 150 (28.4) | 60 (20.8) | 34 (14.6) | *<0.001* |
| **Vital Signs and laboratory findings** |  |  |  |  |
| Heart rate, beats/min | 68.0 ± 12.1 | 68.2 ± 12.1 | 66.6 ± 12.0 | *0.279* |
| Systolic blood pressure, mmHg | 113.9 ± 16.6 | 117.0 ± 18.5 | 119.7 ± 15.6 | *<0.001* |
| BMI, kg/m^2^ | 26.1 ± 4.9 | 27.4 ± 4.9 | 27.7± 5.1 | *<0.001* |
| Overweight/Obese | 253 (54.9) | 167 (64.5) | 146 (66.7) | *0.004* |
| Hemoglobin, g/dl | 13.9 (12.7-15.2) | 14.7 (13.3-15.8) | 14.9 (13.7-15.9) | *<0.001* |
| eGFR, ml/min/1.73 m^2^ |  |  |  |  |
| - < 30 | 31 (5.8) | 13 (4.5) | 2 (0.9) | *0.001* |
| - 30-60 | 200 (37.5) | 91 (31.5) | 69 (29.6) |  |
| - > 60 | 302 (56.7) | 185 (64.0) | 162 (69.5) |  |
| NT-proBNP, pg/ml | 1150.0 (484.0-2637.0) | 850.0 (337.0-1800.0) | 684.0 (292.0-1565.0) | *<0.001* |
| K+, mmol/L | 4.5 (4.2- 4.9) | 4.5 (4.1- 4.8) | 4.4 (4.2- 4.8) | *0.679* |

Results are expressed as n (%), mean ± standard deviation, or median [interquartile range]. Abbreviations: BMI = body mass index; CRT-D = cardiac resynchronization therapy-defibrillator; eGFR = estimated glomerular filtration rate; ICD = implantable cardioverter defibrillator; NT-proBNP = N-terminal pro b-type natriuretic peptide; NYHA = New York Heart Association;

**Supplemental Table 4.** Baseline HFrEF patient characteristics by number of medications.

|  | **None**  **(N=47)** | **Single therapy (N=97)** | **Double therapy**  **(N=261)** | **Triple**  **therapy**  **(N=514)** | **Quadruple therapy**  **(N=801)** | ***p-value*** |
| --- | --- | --- | --- | --- | --- | --- |
| Age, years | 65.5 ± 11.7 | 73.3 ± 11.9 | 72.9 ± 10.9 | 68.9 ± 11.5 | 65.4 ± 11.7 | *<0.001* |
| Female Sex | 8 (17.0) | 19 (19.6) | 56 (21.5) | 89 (17.3) | 151 (18.9) | *0.72* |
| Caucasian | 45 (95.7) | 96 (100.0) | 252 (99.2) | 491 (97.0) | 768 (97.2) | *0.136* |
| Region |  |  |  |  |  |  |
| - Northern Italy | 32 (68.1) | 66 (68.0) | 156 (59.8) | 294 (57.2) | 458 (57.2) | *<0.001* |
| - Centre Italy | 15 (31.9) | 30 (30.9) | 85 (32.6) | 170 (33.1) | 223 (27.8) |  |
| - Southern Italy | 0 | 1 (1.0) | 20 (7.7) | 50 (9.7) | 120 (15.0) |  |
| NYHA |  |  |  |  |  |  |
| - I/II | 38 (80.9) | 75 (77.3) | 197 (75.5) | 409 (79.7) | 652 (81.5) | *<0.001* |
| - III | 6 (12.8) | 19 (19.6) | 64 (24.5) | 102 (19.9) | 142 (17.8) |  |
| - IV | 3 (6.4) | 3 (3.1) | 0 | 2 (0.4) | 6 (0.8) |  |
| Outpatients | 7 (14.9) | 60 (61.9) | 188 (72.0) | 425 (82.7) | 703 (87.8) | *<0.001* |
| Baseline LVEF, % | 32.1 ± 7.1 | 32.3 ± 6.6 | 33.1 ± 6.0 | 31.6 ± 6.6 | 31.0 ± 6.5 | *<0.001* |
| Current smoking | 17 (36.2) | 10 (10.3) | 22 (8.4) | 61 (12.0) | 96 (12.1) | *<0.001* |
| **Clinical history** |  |  |  |  |  |  |
| *De Novo* HF | 42 (89.4) | 35 (36.5) | 43 (16.5) | 58 (11.3) | 72 (9.0) | *<0.001* |
| Type 2 diabetes mellitus | 11 (23.4) | 36 (37.1) | 95 (36.4) | 168 (32.7) | 249 (31.1) | *0.266* |
| Coronary artery disease | 19 (40.4) | 61 (62.9) | 153 (58.6) | 313 (60.9) | 405 (50.8) | *<0.001* |
| Hypertension | 27 (57.4) | 72 (74.2) | 178 (68.5) | 306 (59.9) | 464 (58.1) | *0.002* |
| Atrial fibrillation | 10 (21.3) | 33 (34.0) | 122 (47.3) | 214 (41.7) | 307 (38.4) | *0.004* |
| Chronic kidney disease | 8 (17.4) | 45 (46.4) | 120 (46.2) | 221 (43.2) | 278 (35.2) | *<0.001* |
| Dyslipidemia | 27 (57.4) | 68 (70.8) | 176 (68.0) | 338 (66.4) | 531 (66.6) | *0.599* |
| Dementia | 0 | 0 | 6 (2.3) | 3 (0.6) | 6 (0.8) | *0.092* |
| History of ventricular arrhythmias or ICD shock | 4 (8.5) | 16 (16.5) | 40 (15.3) | 113 (22.0) | 214 (26.8) | *<0.001* |
| ICD or CRT-D | 2 (4.3) | 34 (35.1) | 122 (46.7) | 319 (62.1) | 572 (71.6) | *<0.001* |
| Prior hospitalization in the last 12 months | 6 (12.8) | 14 (14.4) | 58 (22.3) | 131 (25.7) | 204 (25.6) | *0.035* |
| **Vital Signs and laboratory findings** |  |  |  |  |  |  |
| Heart rate, beats/min | 73.3 ± 15.8 | 70.1 ± 12.6 | 70.2 ± 13.8 | 68.7 ± 11.9 | 67.6 ± 12.2 | *0.002* |
| Systolic blood pressure, mmHg | 117.6 ± 17.8 | 124.9 ± 17.7 | 121.5 ± 18.3 | 117.0 ± 16.7 | 114.7 ± 16.8 | *<0.001* |
| BMI, kg/m^2^ | 26.8 ± 6.0 | 26.0 ± 4.9 | 26.4 ± 5.6 | 26.4 ± 4.8 | 26.7 ± 5.1 | *0.733* |
| Overweight/Obese | 25 (55.6) | 44 (50.0) | 135 (57.7) | 253 (57.1) | 420 (58.9) | *0.609* |
| Hemoglobin, g/dl | 13.4 (11.9-14.7) | 13.1 (11.5-14.5) | 13.1 (11.8-14.5) | 13.8 (12.4-15.0) | 14.5 (13.2-15.7) | *<0.001* |
| eGFR, ml/min/1.73 m^2^ |  |  |  |  |  |  |
| - < 30 | 2 (4.3) | 23 (23.7) | 42 (16.1) | 42 (8.2) | 28 (3.5) | *<0.001* |
| - 30-60 | 16 (34.0) | 29 (29.9) | 95 (36.4) | 189 (36.8) | 268 (33.5) |  |
| - > 60 | 29 (61.7) | 45 (46.4) | 124 (47.5) | 283 (55.1) | 505 (63.0) |  |
| NT-proBNP, pg/ml | 1411.5 (583.5-3119.5) | 1363.0 (610.0-4383.0) | 1949.0 (608.0-4606.0) | 1385.0 (482.8-3506.5) | 957.5 (418.3-2100.8) | *<0.001* |
| K+, mmol/L | 4.3 (3.9-4.5) | 4.5 (4.1-4.8) | 4.4 (4.1-4.7) | 4.4 (4.1-4.8) | 4.5 (4.1-4.8) | *0.006* |

Results are expressed as n (%), mean ± standard deviation, or median [interquartile range]. Abbreviations: BMI = body mass index; CRT-D = cardiac resynchronization therapy-defibrillator; eGFR = estimated glomerular filtration rate; ICD = implantable cardioverter defibrillator; NT-proBNP = N-terminal pro b-type natriuretic peptide; NYHA = New York Heart Association;

**Supplemental Table 5.** Comparison between OPTIPHARM-HF and previous registries/trials on female recruitment.

| **HFrEF** | | **HFmrEF/HFpEF** | |
| --- | --- | --- | --- |
|  | Female recruitment |  | Female recruitment |
| *Registries* |  | *Registries* |  |
| OPTIPHARM-HF | 19% | OPTIPHARM-HF | 32% |
| BRING-UP3-HF Study | 16% | BRING-UP3-HF Study | 34% |
| TITRATE-HF | 29% |  |  |
| CHAMP-HF | 29% |  |  |
| CHECK-HF | 34% |  |  |
| SwedeHF | 27% |  |  |
| *Clinical trials* |  | *Clinical trials* |  |
| PARADIGM-HF | 22% | FINEARTS-HF | 46% |
| EMPEROR-REDUCED | 22% | DELIVER | 44% |
| DAPA-HF | 23% | EMPEROR-PRESERVED | 45% |
| VICTOR | 24% | PARAGON-HF | 52% |
| GALACTIC-HF | 21% | TOPCAT-Americas | 50% |
